# Real-Time Imaging of Rabbit Retina with Retinal Degeneration by Using Spectral-Domain Optical Coherence Tomography

**DOI:** 10.1371/journal.pone.0036135

**Published:** 2012-04-27

**Authors:** Yuki Muraoka, Hanako Ohashi Ikeda, Noriko Nakano, Masanori Hangai, Yoshinobu Toda, Keiko Okamoto-Furuta, Haruyasu Kohda, Mineo Kondo, Hiroko Terasaki, Akira Kakizuka, Nagahisa Yoshimura

**Affiliations:** 1 Department of Ophthalmology and Visual Sciences, Kyoto University Graduate School of Medicine, Kyoto, Japan; 2 Center for Anatomical Studies, Kyoto University Graduate School of Medicine, Kyoto, Japan; 3 Department of Ophthalmology, Mie University School of Medicine, Tsu, Japan; 4 Department of Ophthalmology, Nagoya University Graduate School of Medicine, Nagoya, Japan; 5 Laboratory of Functional Biology, Kyoto University Graduate School of Biostudies and Solution Oriented Research for Science and Technology, Kyoto, Japan; Dalhousie University, Canada

## Abstract

**Background:**

Recently, a transgenic rabbit with rhodopsin Pro 347 Leu mutation was generated as a model of retinitis pigmentosa (RP), which is characterized by a gradual loss of vision due to photoreceptor degeneration. The purpose of the current study is to noninvasively visualize and assess time-dependent changes in the retinal structures of a rabbit model of retinal degeneration by using speckle noise-reduced spectral-domain optical coherence tomography (SD-OCT).

**Methodology/Principal Findings:**

Wild type (WT) and RP rabbits (aged 4–20 weeks) were investigated using SD-OCT. The total retinal thickness in RP rabbits decreased with age. The thickness of the outer nuclear layer (ONL) and between the external limiting membrane and Bruch's membrane (ELM–BM) were reduced in RP rabbits around the visual streak, compared to WT rabbits even at 4 weeks of age, and the differences increased with age. However, inner nuclear layer (INL) thickness in RP rabbits did not differ from that of WT during the observation period. The ganglion cell complex (GCC) thickness in RP rabbits increased near the optic nerve head but not around the visual streak in the later stages of the observation period. Hyper-reflective change was widely observed in the inner segments (IS) and outer segments (OS) of the photoreceptors in the OCT images of RP rabbits. Ultrastructural findings in RP retinas included the appearance of small rhodopsin-containing vesicles scattered in the extracellular space around the photoreceptors.

**Conclusions/Significance:**

In the current study, SD-OCT provided the pattern of photoreceptor degeneration in RP rabbits and the longitudinal changes in each retinal layer through the evaluation of identical areas over time. The time-dependent changes in the retinal structure of RP rabbits showed regional and time-stage variations. *In vivo* imaging of RP rabbit retinas by using SD-OCT is a powerful method for characterizing disease dynamics and for assessing the therapeutic effects of experimental interventions.

## Introduction

Retinitis pigmentosa (RP) is an inherited retinal disorder characterized by a progressive loss of visual function due to degeneration of rod and cone photoreceptors and eventual atrophy of the entire retina [1], [2]. However, there are no effective treatments for RP. Various animal models of RP have been developed and studied to elucidate the pathophysiology of the disease and to develop new treatments [3]–[10]. Of these models, only monkeys have a macula, an important area for vision due to the high density of cone photoreceptors. However, it is not easy to study the pathophysiology of RP in monkeys due to handling and breeding difficulties. Rabbits are known to have a visual streak, where the rod and cone photoreceptor density is highest, about 3 mm ventral to the optic nerve head (ONH) [11], [12]. Rabbits are easy to breed and handle, and the physiology and morphology of rabbit retina is well understood [11]–[14]. Additionally, in mid-sized animals like rabbits, surgical treatments such as subretinal injection of cells for regenerative therapy [15], [16], vectors for gene therapy [17], and implantation of intraocular devices [18], [19] are easily performed. Therefore, rabbits are very useful for studying retinal diseases and testing new therapeutic interventions. For these reasons, we used transgenic (Tg) rabbits with mutated rhodopsin (Pro 347 Leu, RP rabbits) as a mid-sized model for RP [20] to study the pathophysiology and develop new evaluation systems for retinal degeneration.

Optical coherence tomography (OCT) devices allow non-invasive detection of retinal architecture, including quantitative measurements of retinal thickness and longitudinal observation of the retinal architecture [21]. The technological advances in spectral-domain OCT (SD-OCT) have enabled high-speed scanning and improved image resolution [22]. Furthermore, the exact averaging of B-scans with a three-dimensional eye-tracking system and high-speed scanning have enabled sufficient reduction in speckle noise, the most influential artificial noise that blurs the boundaries between retinal layers [23], [24]. These advances have improved visualization of individual retinal layers, including both the outer retina and the inner retina (i.e., ganglion cell layer and inner plexiform layer [IPL] in humans) [25], [26]. SD-OCT imaging also enables evaluation of the junction between the inner segment (IS) and the outer segment (OS) of the photoreceptors (IS/OS) [27]–[29] and that of the external limiting membrane (ELM) [30], [31] as hallmarks of photoreceptor integrity. That is, visual function can be speculated from OCT images to some extent. Thus, the use of OCT imaging in humans has contributed to a more detailed understanding of the pathophysiology of many retinal diseases. In mice, the retina has been clearly visualized using SD-OCT [32]–[37]. Thus, in experimental animals, SD-OCT may allow *in vivo* detection and monitoring of changes in retinal architecture without sacrificing animals.

In mouse models of retinal degeneration, Fischer [36] and Huber et al. [32] detected and analyzed photoreceptor degeneration by using SD-OCT. They imaged the thinning of inner retinal layers and compared the total retinal thickness with that of normal mice in several mouse RP models. Yamauchi et al. reported the retinal architecture of rabbits by using SD-OCT following iodoacetic acid-induced photoreceptor degeneration [38]. However, retinal pathomorphology of genetically engineered rabbit models of RP, which mimic human RP [20], and longitudinal assessment of changes in the individual retinas remain to be studied with SD-OCT.

The purpose of this study was to visualize the time-dependent changes in photoreceptors, elucidate the pattern of changes in each retinal layer around the visual streak in identical eyes of RP rabbits by using SD-OCT, and assess the visual functions by electroretinography (ERG).

## Results

### Visualization of retinal structures in RP rabbits with SD-OCT

**Figure 1:**
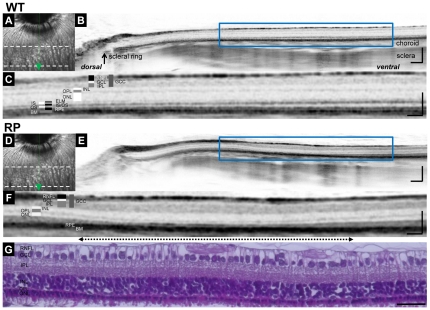
SD-OCT images of WT and Retinitis Pigmentosa (RP) rabbit retinas and histology of the visual streak in an RP rabbit. (A) A fundus infrared image of a WT rabbit retina, including optic nerve head (ONH) and visual streak. The area between dotted lines is the visual streak. (B) A vertical SD-OCT image along the green arrow in panel A, which passes through the center of the ONH. On this vertical image, the scleral ring was regarded as the lower margin of the ONH. (C) A magnified OCT image of the area enclosed by the blue square in panel B, which includes the visual streak. (D) A fundus infrared image of a RP rabbit retina, including the ONH and visual streak. (E) A vertical SD-OCT image of a 20-week-old RP rabbit along the green arrow in panel D. (F) A magnified OCT image of the area enclosed by the blue square in panel E. The 2.2 mm width of this OCT section was vertically cut between 1.8 mm and 4.0 mm ventral to the inferior edge of the ONH. A dotted arrow indicates the region of the visual streak. (G) Hematoxylin-Eosin staining of a retinal section corresponding to the area in the OCT image in F. Scale Bar = 200 µm (B, E), 100 µm (C, F), and 50 µm (G). RNFL, retinal nerve fiber layer; GCL, ganglion cell layer; IPL, inner plexiform layer; GCC, ganglion cell complex; INL, inner nuclear layer; OPL, outer plexiform layer; ONL, outer nuclear layer; ELM, external limiting membrane; IS, inner segments of photoreceptors; OS, outer segments of photoreceptors; IS/OS, junctions between IS and OS; RPE, retinal pigment epithelium; and BM, Bruch's membrane.

We first investigated whether the retinal structures of WT rabbits could be clearly visualized using SD-OCT. Vertical OCT images, which passed through the center of the ONH (Fig. 1A), permitted clear identification of each retinal layer, the choroid, and sclera of WT rabbits (Figs. 1B and 1C). The ELM and IS/OS lines were also clearly identifiable, the integrity of which have been shown to be positively associated with visual function. In the vertical OCT images, the scleral ring was defined as the edge of the ONH so that OCT measurements could be longitudinally compared between each rabbit and between WT and RP rabbits (Fig. 1B).

Next, we examined a 20-week-old RP rabbit that expressed mutated rhodopsin (Figs. 1D–1F). The outer nuclear layer (ONL) of the RP rabbit was much thinner than the WT rabbit. Furthermore, in the RP rabbit, the photoreceptors around the visual streak (indicated by the dotted arrow), where the densities of rod and cone photoreceptors were the highest, appeared to be more severely damaged than in any other area. In this area, the ONL was very thin and the outer plexiform layer (OPL) was faint or absent depending on the distance from the ONH and the IS/OS line was undetectable (Fig. 1F). This regional variation in photoreceptor damage was also detected with hematoxylin and eosin (H&E) staining in the same eye (Fig. 1G).

### Time-dependent changes in the photoreceptor layers and in the visual function of RP rabbits

**Figure 2:**
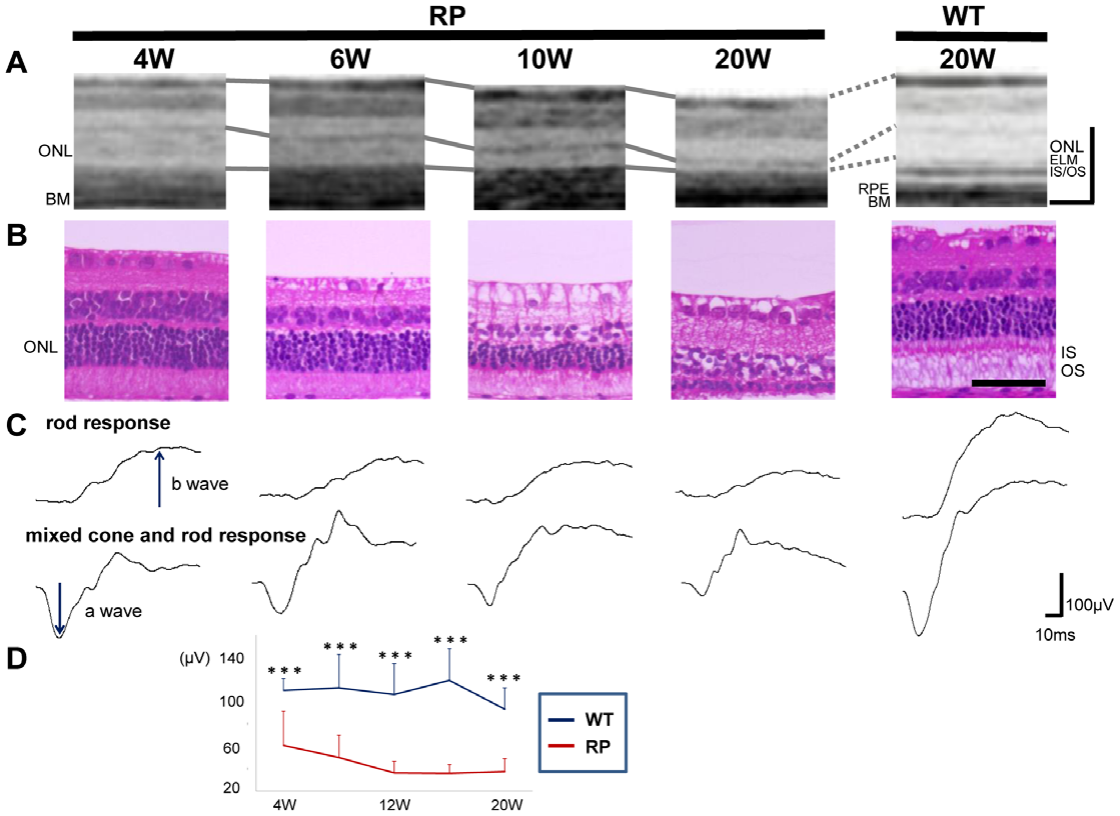
Time-dependent changes in morphological features of the retina and visual function in the RP rabbits. (A) SD-OCT images beneath the visual streak in an RP rabbit at 4, 6, 10, and 20 weeks and in a 20-week-old WT rabbit. The total retinal and ONL thickness in the RP rabbits decreased with age. The IS and OS were highly reflective in the RP rabbits compared with the WT rabbits. ONL, outer nuclear layer; and OS, outer segments of photoreceptors. (B) Hematoxylin-eosin staining of retinas in 4-, 6-, 10-, and 20-week-old RP and 20-week-old WT rabbits. The ONL in RP rabbits thinned with age. In 20-week-old RP rabbits, only 1–2 layers of nuclei were detected in the ONL. (C) Representative scotopic electroretinograms of 4-, 6-, 10-, and 20-week-old RP and 20-week-old WT rabbits. (D) The a-wave amplitude of the mixed rod and cone response. The amplitude was smaller in the RP rabbits than in the WT rabbits. The differences between the WT and RP rabbits were significant at all study points between 4 and 20 weeks. *P<0.05, ***P<0.001 (unpaired *t*-test). Scale Bar = 100 µm in A, and 50 µm in B. ONL, outer nuclear layer; ELM, external limiting membrane; IS/OS, junctions between inner segment (IS) and outer segment (OS); RPE, retinal pigment epithelium; and BM, Bruch's membrane.

As observations revealed that photoreceptor damage was severe around the visual streak, we were encouraged to investigate the time-dependent changes in the photoreceptors of identical RP rabbits beneath the visual streak with SD-OCT and compared them with those of the WT rabbits (Fig. 2A). At 4 weeks of age (with the youngest that can be examined by OCT), the ONL of RP rabbits was almost as thick as WT rabbits. Following 4 weeks of age, the ONL thickness in RP rabbits decreased. At 20 weeks, the ONL thickness in RP rabbits was much smaller than in WT rabbits. Photoreceptor IS and OS, where visual phototransduction occurs, were thin in RP rabbits. In contrast, the architecture of the inner retina was relatively preserved in RP rabbits at both 10 and 20 weeks of age.

In the current SD-OCT study, there were additional findings in the photoreceptor layers. In the sections of WT rabbits, the reflectivity of IS and OS was low compared to that of the ELM and IS/OS lines. In contrast, the IS and OS were highly reflective in RP rabbits, and almost equivalent to the ELM and IS/OS lines throughout the study ages (Fig. 2A).

To compare the SD-OCT data with those from the histological examination, histological sections of the age-matched RP and WT rabbits were prepared (Fig. 2B). The number of photoreceptors and thickness of the ONL, IS, and OS in the RP rabbits decreased with age, which is consistent with those of a previous report [20]. At 20 weeks of age, the nuclei of photoreceptors in RP rabbits were reduced to 1 or 2 rows, which was much less compared to WT rabbits. The magnitude of the decrease in ONL thickness appears similar between the histological and SD-OCT data (Figs. 2A and 2B). In the histological sections of a 4-week-old RP rabbit, the total retinal thickness and the ONL thickness were almost the same as those of the WT rabbit, and the IS and OS appeared intact. The high reflectivity in the IS and OS observed in the OCT sections was difficult to explain by the histological sections (Figs. 2A and 2B).

Next, to evaluate visual function of the rod and cone systems of RP rabbits, scotopic full-field ERG was recorded (Fig. 2C). The a-wave of the mixed cone and rod response, which mainly originates from the photoreceptors, was smaller in RP rabbits (61.2±30.5 µV) (mean ± SD) than in WT rabbits (110.3±10.7 µV; *P* = 0.010, unpaired *t*-test) as early as 4 weeks. The a-wave amplitude was reduced with RP rabbit aged (Fig. 2D). At the age of 20 weeks, the a-wave amplitude decreased to 37.6±11.5 µV in RP rabbits and was significantly less than that of WT rabbits (93.5±19.0 µV; *P*<0.001, unpaired *t*-test, Figs. 2C and 2D). The b-wave amplitude of the rod response, which originates indirectly from bipolar and Müller cells, was 97.3±33.2 µV in RP rabbits and was less than that of WT rabbits (280.8±71.3 µV; *P*<0.001, unpaired *t*-test, Fig. 2C). These data suggest that the visual function of both the rod and cone systems was disturbed in RP rabbits, consistent with a previous report [20]. These results indicate that loss of photoreceptors and concomitant visual dysfunction gradually occurs in RP rabbits.

### Vesicles cleaved from photoreceptors and disorganization of IS and OS in RP rabbits account for the hyper-reflectivity seen in SD-OCT images

**Figure 3:**
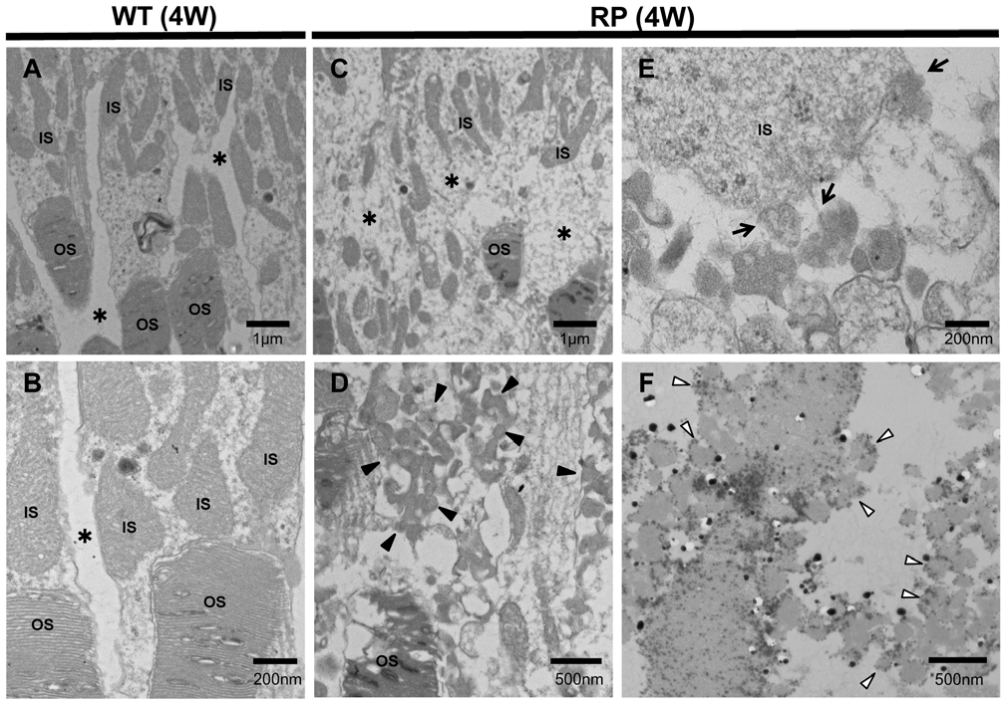
Ultrastructure of photoreceptors in WT and RP rabbits. (A, B) Ultrastructure of photoreceptors in 4-week-old WT rabbits. The inner (IS) and outer segments (OS) of the photoreceptors were regular and dense. There are no vesicles in the extracellular spaces (*). (C–E) Ultrastructure of the photoreceptors in the 4-week-old RP rabbits. The IS and OS were less organized than those in the WT rabbits. In the magnified image (D), the RP rabbit retina showing many small vesicles (arrowheads) accumulated in the extracellular spaces (indicated with * in panel C). The vesicles appeared to be cleaved from the IS into the extracellular space around the photoreceptors (arrows in panel E). (F) Ultrastructural immunohistochemistry by using an anti-rhodopsin antibody. The small vesicles (disintermediated arrowheads) in the extracellular spaces around the photoreceptors exhibit black dots indicating the presence of rhodopsin.

To elucidate the cause of the hyper-reflective change in the outer photoreceptor layers of RP rabbits in SD-OCT sections, we examined and compared the ultrastructure of the retina between RP and WT rabbits at 4 or 20 weeks of age. In WT rabbits, the IS and OS exhibited a dense and regular arrangement (Figs. 3A, 3B, S1A and S1B). In contrast, in the RP retinas, the IS and OS were less organized at 4 weeks of age (Figs. 3C and 3D), and they were mostly absent at 20 weeks of age (Figs. S1C and S1D). Magnified images of the RP retinas revealed large number of small, approximately 100 nm, vesicles scattered in the extracellular space around the photoreceptors (arrowheads in Fig. 3D and S1D). These small vesicles appeared to be cleaved from the membrane of the IS in RP rabbits (arrows in Fig. 3E and S1D).The disrupted organization and the presence of vesicles between the IS and OS on ultra microscopy may account for the hyper-reflectivity seen in the corresponding area of the SD-OCT images.

To determine the origin of the vesicles, we performed ultrastructural immunohistochemistry by using monoclonal antibodies against rhodopsin (Fig. 3F). In RP retinas, numerous vesicles with dots were observed, indicating the presence of rhodopsin within the vesicles (disintermediated arrowheads in Fig. 3F).

### Time-dependent changes in the individual retinal layers in SD-OCT sections exhibit regional and time-stage variations in RP rabbits

**Figure 4:**
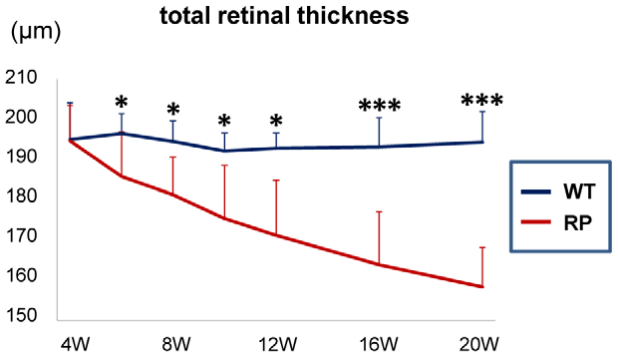
Time-dependent changes in total retinal thickness, and mixed cone and rod response in the WT and RP rabbits. The total retinal thicknesswas measured within a circle 1 mm in diameter 3 mm ventral to the lower margin of the ONH and averaged. The total retinal thickness in the WT rabbits (indicated with a blue line) was unchanged during observation, whereas that in the RP rabbits (indicated with a red line) severely decreased with age. *P<0.05, ***P<0.001 (unpaired *t*-test).

In the SD-OCT examinations, the retinal thickness in the RP rabbits appeared to decrease with age. Therefore, we quantitatively measured the mean total retinal thickness around the visual streak in the WT and RP rabbits (Figs. 4A and S2). As shown in Fig. 4A, the total retinal thickness in WT rabbits did not change with age, whereas that of the RP rabbits progressively decreased. The total retinal thickness in WT and RP rabbits was not significantly different at 4 weeks. However, after 6 weeks, the differences in the total retinal thickness increased and continued with age. At 20 weeks, the total retinal thickness in RP rabbits was 165.8±8.5 µm and significantly smaller than that of WT rabbits (194.3±7.7 µm; *P*<0.001, unpaired *t*-test).

OCT examination showed that the photoreceptors were most severely damaged at the visual streak, approximately 3 mm ventral to the ONH [11]. Therefore, we longitudinally examined regional and periodical variations in the progression of retinal degeneration in RP rabbits. For this purpose, we measured the thickness of each retinal layer within 0.5-mm areas 4 mm ventral to the lower edge of the ONH as a function of distance from the lower optic disc margin at 4, 6, 10, and 20 weeks by using the vertical OCT images that passed through the center of the ONH and visual streaks (Fig. S3).

#### ONL thickness

**Figure 5:**
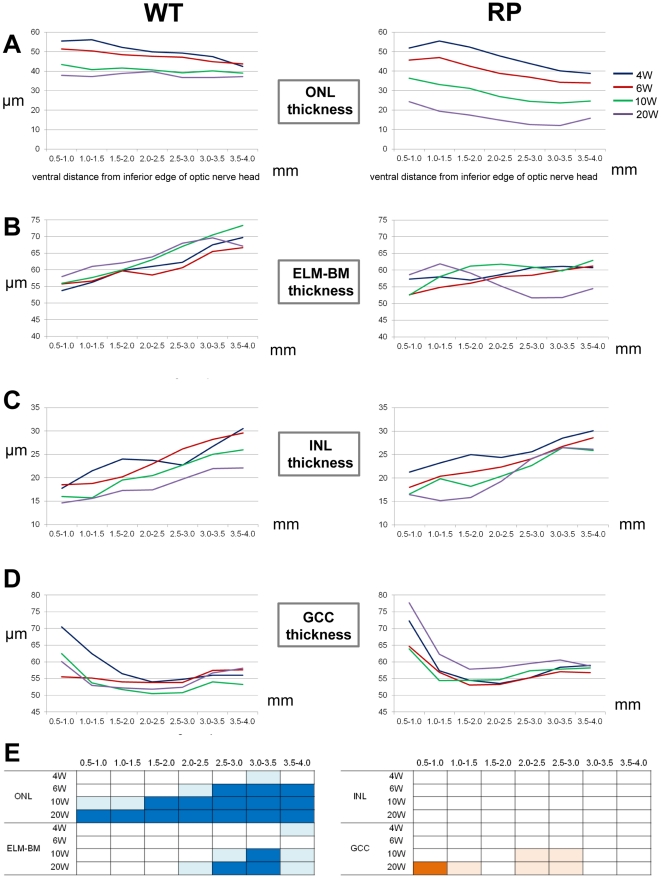
Time-dependent changes in the thickness of individual retinal layers in WT and RP rabbits. (A–D) Time-dependent changes in ONL (A), ELM–BM (B), INL (C), and GCC (D) thickness measured with vertical OCT sections of WT and RP rabbits. Mean values at 4, 6, 10, and 20 weeks are indicated with blue, red, green, and purple lines, respectively. X-axes indicate the distance from the inferior edge of the ONH. (E) A schema to show time course and regional variations in the thickness of each retinal layer in RP rabbits from 4 to 20 weeks. Blue color indicates the regions where retinal thickness of RP rabbits was significantly smaller than that of WT rabbits. Orange color indicates the regions where retinal thickness of RP rabbits was significantly larger than that of WT rabbits. Deep and light colors indicate *P*<0.001 and *P*<0.05, respectively (unpaired *t*-test). ONL, outer nuclear layer; ELM, external limiting membrane; BM, Bruch's membrane; INL, inner nuclear layer; and GCC, ganglion cell complex.

We first evaluated the thickness of the ONL where the nuclei of photoreceptors are located (Fig. 5A). In WT rabbits, the ONL in each area became slightly thinner with age. In younger WT rabbits (4–6 weeks old), the ONL was thinner in areas more distant from the ONH. In RP rabbits, the decrease in ONL thickness with age was more progressive than that of WT rabbits. At any age examined, thinning of the ONL was greater in areas more distant from the ONH. At 10 and 20 weeks, the ONL was thinnest in the area 3.0–3.5 mm ventral to the ONH.

At 4 weeks, the ONL thickness in RP rabbits was significantly less than that of WT rabbits in only the area 3.0–3.5 mm from the ONH (*P* = 0.037, unpaired *t*-test). Areas that exhibited a difference in ONL thickness between WT and RP rabbits expanded with age. At 20 weeks, the ONL thickness in RP rabbits was significantly smaller than that of WT rabbits in each of the 7 areas examined (*P*<0.001, unpaired *t*-test, Fig. 5E).

#### ELM–BM thickness

The ELM–BM thickness was evaluated because the area between the ELM and BM includes the length of the IS and OS where visual phototransduction occurs (Figs. 5B and S3). In WT rabbits, the ELM–BM thickness was larger in areas more distant from the ONH at any age examined. On the other hand, in RP rabbits, the differences in the ELM–BM thickness between areas as a function of distance from the ONH were smaller compared to those of WT rabbits at 4–10 weeks; the ELM–BM thickness appeared to decrease mainly in the areas distant from the optic disc. In 20-week-old RP rabbits, the ELM–BM thickness markedly decreased in the area 2.5–3.5 mm ventral to the ONH and was significantly less than that in the corresponding areas in WT rabbits (*P*<0.001, unpaired *t*-test, Fig. 5E).

#### INL thickness

The INL comprises the nuclei of bipolar, horizontal, amacrine, and Müller cells. The INL thickness was larger in areas more distant from the ONH in both WT and RP rabbits at the ages of 4 to 20 weeks (Fig. 5C). The INL thickness in WT and RP rabbits was not significantly different in each corresponding area at all the ages examined (Fig. 5E).

#### GCC thickness

The GCC consists of the retinal nerve fiber layer (axons of ganglion cells), ganglion cell layer (somas of ganglion cells), and IPL. To determine the influence of photoreceptor degeneration on the inner retina, GCC thickness was measured (Figs. S3 and 5D). The GCC thickness in the WT and RP rabbits exhibited a similar pattern in all the areas examined at the ages of 4 and 6 weeks. However, in 20-week-old RP rabbits, the GCC thickness in the areas close to the ONH was larger than in younger RP rabbits and in the corresponding areas of 20-week-old WT rabbits (*P*<0.001 for both, unpaired *t*-test, Fig. 5E).

In summary, the decrease in the ONL and ELM–BM thickness in RP rabbits was first detected in the areas approximately 3.0 mm ventral to the lower edge of the ONH (areas corresponding to the visual streak). Thinning of the IS and OS (measured as the ELM–BM thickness) followed thinning of the ONL. In contrast, the INL thickness in RP rabbits did not change throughout the observational period of 4 to 20 weeks. The GCC thickness in RP rabbits increased in areas away from the visual streaks but close to the ONH in the later phase of observation (Fig. 5E).

## Discussion

In this study, we examined time-dependent changes in photoreceptor degeneration in identical RP rabbits, and compared the pattern of changes in individual retinal layers between WT and RP rabbits for the first time by using SD-OCT. In RP rabbits, we observed regional differences in the degree of photoreceptor loss. That is, the ONL (ONL: the somas of photoreceptors) in RP rabbits was thinnest beneath the visual streak, where the densities of rod and cone photoreceptors were the highest in WT rabbits, and the photoreceptors of RP rabbits were relatively preserved in the area near the ONH. The current observations by using SD-OCT revealed longitudinal changes in the RP rabbit retina that were fairly consistent with a previous histological study of the RP rabbits [20] and reports based on other animal models of RP [3], [9].

To elucidate the unique “highly reflective IS and OS" feature of the outer photoreceptor layer during photoreceptor degeneration in RP rabbits, an electron microscopy study was conducted on 4- or 20-week-old RP rabbits. We detected vesicles around the photoreceptors and loss of most of the IS and OS. We speculate that these destructive structures in RP rabbits cause the hyper-reflectivity seen in the outer photoreceptor layers (between ELM and BM) on SD-OCT images (Fig. 2A).The vesicles appeared to be cleaved from the IS, as described in a previous report [20]. Moreover, these vesicles were shown to include rhodopsin by ultrastructural immunohistochemistry (Fig. 3F), indicating that the particles were derived from photoreceptors. In SD-OCT images of 4-week-old RP rabbits, the area between the ELM and BM was hyper-reflective even though the reflectivity and the thickness of the ONL were unchanged (Fig. 2A). These observations point to the mechanism by which photoreceptors degenerate in RP rabbits. That is, defective transport of rhodopsin from the IS to the OS, which was demonstrated in mice with mutated rhodopsin P347S by using an antibody against the mutated rhodopsin [39], is followed by cleavage of vesicles from the IS, and finally cell bodies of photoreceptors degenerate. It is speculated that early stage RP patients may have mutations in the rhodopsin gene if hyper-reflective patterns are detected with SD-OCT in the area corresponding to the IS and OS, though further studies are needed to confirm this speculation.

Aleman et al. reported the following disease sequence in human and murine RP caused by mutation of the rhodopsin gene: ONL diminution with INL thickening, amalgamation of residual ONL with the thickened INL, and progressive retinal remodeling with eventual thinning seen in OCT [40]. In our SD-OCT study, the INL thickness was not significantly different between WT and RP rabbits at the ages of 4 to 20 weeks. In contrast, the GCC thickness in RP rabbits paradoxically increased in the later phase of observation. Previous studies have suggested that the increase in the INL/inner retinal thickness in patients with RP maybe related to Müller glial activation with hypertrophy [40]–[42]. In the current study with RP rabbits, the observational period may be too short to detect an increase in INL thickness, or the gliosis of Müller cells may occur preferably within the GCC than in the INL.

This study has some limitations. The area imaged with SD-OCT was quite restricted such that the degeneration in RP retinas obtained with SD-OCT did not always correlate with the total retinal function. Furthermore, OCT or ERG could not be performed on rabbits younger than 4 weeks as their eyelids had not yet opened.

In conclusion, despite these short comings, *in vivo* time-dependent changes in the retinal structures were seen layer-by-layer in RP rabbits by using SD-OCT. These changes in the retinal structure had regional and temporal variations not only in the outer retina but also in the inner retina of RP rabbits. This study demonstrates that *in vivo* imaging with SD-OCT can facilitate the characterization of morphological disease dynamics and serve as a powerful tool for developing new treatments, such as gene therapy, intraocular devices, and neuroprotective treatments, in rabbit models of RP.

## Methods

### Experimental animals

This study was conducted in accordance with the Association Research in Vision and Ophthalmology (ARVO) Statement for the Use of Animals in Ophthalmic and Vision Research. All the protocols were approved by the Institutional Review Board of the Kyoto University Graduate School of Medicine (MedKyo11229).

New Zealand White rabbits (NZW, WT) and RP rabbits with rhodopsin P347L mutation (NZW, RP) [20] were purchased from Kitayama Labes Co., Ltd (Ina, Nagano, Japan). All rabbits were kept under a 14 h–10 h light-dark cycle (approximately 200 lux), given free access to water, and fed once a day. For the ERG recording and SD-OCT image acquisition, male WT (n = 4–10 eyes) and RP rabbits (n = 10–16 eyes) were used.

### Retinal histology

Rabbit eyes were fixed overnight in a mixture of 10% neutral buffered formalin and 2.5% glutaraldehyde and then transferred to 10% neutral buffered formalin. The tissues were trimmed, embedded in paraffin, sectioned vertically through the optic nerve (superior-inferior), and stained with hematoxylin and eosin. The retina beneath the visual streak was examined and compared between 10- and 20-week-old WT and RP rabbits.

### ERG

ERG was performed to assess the visual function of WT and RP rabbits at 4, 6, 10, and 20 weeks. Pupils were dilated with tropicamide (0.5%) and phenylephrine (0.5%) eye drops. Rabbits were dark-adapted for more than 60 min before anesthetization with an intramuscular injection of ketamine (25 mg/kg) and xylazine (2 mg/kg). ERG was recorded using a gold loop corneal electrode with a light-emitting diode (Mayo Corp., Inazawa, Japan). A reference electrode was placed in the mouth, and a ground electrode was attached to the ear. Stimuli were produced with a light-emitting diode stimulator (Mayo Corp.). The ERG response signals were amplified, digitized at 10 kHz with a band-pass filter of 0.3 to 500 Hz and analyzed (PowerLab 2/25; AD instruments, New South Wales, Australia). Two steps of stimulus intensities (ISCEV standard; scotopic 0.01 and scotopic 3.0) were used for evaluating rod and mixed cone and rod responses. The b-wave amplitude of the rod response and the a-wave amplitude of the mixed cone and rod response were analyzed.

### SD-OCT

After ERG recording, rabbits were placed on a platform such that the visual streaks, which were approximately 3 mm ventral to the ONH, were located at the center of the image. The SD-OCT machine used in this study was *Multiline* OCT (Heidelberg Engineering, Heidelberg, Germany), which was customized based on a Spectralis HRA+OCT [37]. The *Multiline* OCT uses an 870-nm super-luminescent diode as a light source. The scan rate of the SD-OCT was 47,000 A-scans per second, with an axial resolution of ∼7 µm.

### Measurement and evaluations of total retinal thickness by using SD-OCT

To measure the total retinal thickness at the visual streak, a volume scan image was obtained (Fig. S2A). The lines of the vitreoretinal interface and BM were manually delineated at each horizontal section in a masked fashion (Figs. S2B and S2C). The mean total retinal thickness was measured within a red circle 1 mm in diameter, the center of which was 3 mm ventral to the inferior edge of the ONH, as determined by the software supplied by Heidelberg Engineering (Figs. S2D and S2E).

### Measurements and evaluation of the thickness of retinal layers on vertical SD-OCT images

To measure and assess the thickness of each retinal layer, vertical OCT images, which passed through the center of the ONH and included the visual streak, were obtained by averaging 100 B-scans. To measure the thickness of the ONL, ELM–BM, INL, and GCC, the boundary lines between the OPL and ONL, ELM and BM, IPL and INL, and the vitreoretinal interface and IPL were manually delineated in a masked fashion (Fig. S3). The thickness of each retinal layer within the areas (0.5 mm each) 4 mm ventral to the lower edge of the ONH was measured as a function of distance from the lower optic disc margin by using the software supplied by Heidelberg Engineering (Fig. S3).

### Electron Microscopy

The enucleated rabbit eyes were fixed in the same manner as the H&E stain. The eyes were subsequently fixed in 1% osmium tetroxide for 90 min. The retina was dehydrated through a graded series of ethanol (50–100%), cleared in propylene oxide, and embedded in epoxy resin. Ultrathin sections were cut by using an ultramicrotome and stained with uranyl acetate and lead citrate. For ultrastructural immunohistochemistry, the enucleated RP rabbit eyes were fixed in 4% paraformaldehyde and 0.05% glutaraldehyde for 4 h. The fixed retina was cut with a microslicer (Microslicer DTK-1000, Dosaka EM, Kyoto, Japan) into sections with a thickness of 65 µm. The sections were incubated with mouse monoclonal anti-rhodopsin antibody (Ret-P1 (sc-57433), Santa Cruz, California, U.S.A.) and subsequently, with gold-conjugated Fab fragment of goat anti-mouse IgG (Nanogold, Molecular Probes, Inc., Oregon, U.S.A.), followed by silver enhancement (HQ Silver, Nanoprobes, Inc., New York, U.S.A.). The stained sections were observed by transmission electron microscopy (H-7650, Hitachi Co., Tokyo, Japan).

### Statistical analysis

Data from WT and RP rabbits were analyzed with an unpaired *t*-test by using PASW Statistics version 18.0 (SPSS Inc., Chicago, IL). The level of statistical significance was set at *P*<0.05.

## Supporting Information

Figure S1 **Ultrastructure of photoreceptors in 20-week-old WT and RP rabbits.** (A, B) Ultrastructure of photoreceptors in 20-week-old WT rabbits. The inner segments of photoreceptors (IS) and the outer segments of photoreceptors (OS) were regular and dense. (C, D) Ultrastructural changes in 20-week-old RP rabbits. The IS and OS were mostly absent, and the residual IS and OS were less organized than those in WT rabbits. In the magnified image (D), many small vesicles (arrowheads) appeared to be cleaved from the IS into the extracellular space around the photoreceptors (arrows).
(TIF)

Figure S2 **Measurement of mean total retinal thickness.** (A) An infrared image on volume scan mode of SD-OCT. In the volume scan mode, the region ventral to the optic disc, including visual streak (19 lines in vertical 15°× horizontal 30°) was imaged. (B) One of the 19 horizontal OCT sections on volume scan mode. The lines of the vitreoretinal interface and the Bruch's membrane are manually delineated at each horizontal section (C). (D) The retinal thickness map constructed from the volume scan OCT images. Total retinal thickness was measured within the red circle shown (E). The diameter of the red circle was 1 mm, and the center was 3 mm ventral to the inferior edge of the ONH (D, E).
(TIF)

Figure S3 **Measurement of the thickness of individual retinal layers.** Four vertical OCT sections that pass through the center of the ONH and visual streak are shown. On each section, the boundary lines between each retinal layer were manually delineated. The ONL, ELM–BM, INL, and GCC thicknesses were evaluated in 0.5-mm segments as a function of the distance from the inferior optic disc margin up to 4.0 mm ventral to the inferior edge of the ONH. ONL, outer nuclear layer; ELM, external limiting membrane; BM, Bruch's membrane; INL, inner nuclear layer; and GCC, ganglion cell complex.
(TIF)

## References

[1] Hartong DT, Berson EL, Dryja TP (2006). Retinitis pigmentosa.. Lancet.

[2] Mendes HF, van der Spuy J, Chapple JP, Cheetham ME (2005). Mechanisms of cell death in rhodopsin retinitis pigmentosa: implications for therapy.. Trends Mol Med.

[3] Petters RM, Alexander CA, Wells KD, Collins EB, Sommer JR (1997). Genetically engineered large animal model for studying cone photoreceptor survival and degeneration in retinitis pigmentosa.. Nat Biotechnol.

[4] Narfström K (1983). Hereditary progressive retinal atrophy in the Abyssinian cat.. J Hered.

[5] Chader GJ (2002). Animal models in research on retinal degenerations: past progress and future hope.. Vision Res.

[6] Petersen-Jones SM (1998). Animal models of human retinal dystrophies.. Eye (Lond).

[7] Barnett KC, Curtis R (1985). Autosomal dominant progressive retinal atrophy in Abyssinian cats.. J Hered.

[8] Menotti-Raymond M, David VA, Schäffer AA, Stephens R, Wells D (2007). Mutation in CEP290 discovered for cat model of human retinal degeneration.. J Hered.

[9] Kijas JW, Cideciyan AV, Aleman TS, Pianta MJ, Pearce-Kelling SE (2002). Naturally occurring rhodopsin mutation in the dog causes retinal dysfunction and degeneration mimicking human dominant retinitis pigmentosa.. Proc Natl Acad Sci U S A.

[10] Ng YF, Chan HH, Chu PH, To CH, Gilger BC (2008). Multifocal electroretinogram in rhodopsin P347L transgenic pigs.. Invest Ophthalmol Vis Sci.

[11] Famiglietti EV, Sharpe SJ (1995). Regional topography of rod and immunocytochemically characterized “blue" and “green" cone photoreceptors in rabbit retina.. Vis Neurosci.

[12] Rockhill RL, Daly FJ, MacNeil MA, Brown SP, Masland RH (2002). The diversity of ganglion cells in a mammalian retina.. J Neurosci.

[13] Marc RE (1986). Neurochemical stratification in the inner plexiform layer of the vertebrate retina.. Vision Res.

[14] Vaney DI, Young HM, Gynther IC (1991). The rod circuit in the rabbit retina.. Vis Neurosci.

[15] Osakada F, Hirami Y, Takahashi M (2009). Stem cell biology and cell transplantation therapy in the retina.. Biotechnol Genet Eng Rev.

[16] Stanzel BV, Liu Z, Brinken R, Braun N, Holz FG (2012). Subretinal delivery of ultrathin rigid-elastic cell carriers using a metallic shooter instrument and biodegradable hydrogel encapsulation.. Invest Ophthalmol Vis Sci.

[17] Acland GM, Aguirre GD, Ray J, Zhang Q, Aleman TS (2001). Gene therapy restores vision in a canine model of childhood blindness.. Nat Genet.

[18] Tao W, Wen R, Goddard MB, Sherman SD, O'Rourke PJ (2002). Encapsulated cell-based delivery of CNTF reduces photoreceptor degeneration in animal models of retinitis pigmentosa.. Invest Ophthalmol Vis Sci.

[19] Bush RA, Lei B, Tao W, Raz D, Chan CC (2004). Encapsulated cell-based intraocular delivery of ciliary neurotrophic factor in normal rabbit: dose-dependent effects on ERG and retinal histology.. Invest Ophthalmol Vis Sci.

[20] Kondo M, Sakai T, Komeima K, Kurimoto Y, Ueno S (2009). Generation of a transgenic rabbit model of retinal degeneration.. Invest Ophthalmol Vis Sci.

[21] Wojtkowski M, Bajraszewski T, Gorczynska I, Targowski P, Kowalczyk A (2004). Ophthalmic imaging by spectral optical coherence tomography.. Am J Ophthalmol.

[22] Chen TC, Cense B, Pierce MC, Nassif N, Park BH (2005). Spectral domain optical coherence tomography: ultra-high speed, ultra-high resolution ophthalmic imaging.. Arch Ophthalmol.

[23] Sakamoto A, Hangai M, Yoshimura N (2008). Spectral-domain optical coherence tomography with multiple B-scan averaging for enhanced imaging of retinal diseases.. Ophthalmology.

[24] Hangai M, Yamamoto M, Sakamoto A, Yoshimura N (2009). Ultrahigh-resolution versus speckle noise-reduction in spectral-domain optical coherence tomography.. Opt Express.

[25] Byeon SH, Chu YK, Lee H, Lee SY, Kwon OW (2009). Foveal ganglion cell layer damage in ischemic diabetic maculopathy: correlation of optical coherence tomographic and anatomic changes.. Ophthalmology.

[26] Nakano N, Hangai M, Nakanishi H, Mori S, Nukada M (2011). Macular Ganglion Cell Layer Imaging in Preperimetric Glaucoma with Speckle Noise-Reduced Spectral Domain Optical Coherence Tomography.. Ophthalmology.

[27] Sandberg MA, Brockhurst RJ, Gaudio AR, Berson EL (2005). The association between visual acuity and central retinal thickness in retinitis pigmentosa.. Invest Ophthalmol Vis Sci.

[28] Costa RA, Calucci D, Skaf M, Cardillo JA, Castro JC (2004). Optical coherence tomography 3: Automatic delineation of the outer neural retinal boundary and its influence on retinal thickness measurements.. Invest Ophthalmol Vis Sci.

[29] Chen TC, Cense B, Miller JW, Rubin PA, Deschler DG (2006). Histologic correlation of in vivo optical coherence tomography images of the human retina.. Am J Ophthalmol.

[30] Oishi A, Hata M, Shimozono M, Mandai M, Nishida A (2010). The significance of external limiting membrane status for visual acuity in age-related macular degeneration.. Am J Ophthalmol.

[31] Murakami T, Nishijima K, Sakamoto A, Ota M, Horii T (2011). Association of pathomorphology, photoreceptor status, and retinal thickness with visual acuity in diabetic retinopathy.. Am J Ophthalmol.

[32] Huber G, Beck SC, Grimm C, Sahaboglu-Tekgoz A, Paquet-Durand F (2009). Spectral domain optical coherence tomography in mouse models of retinal degeneration.. Invest Ophthalmol Vis Sci.

[33] Kim KH, Puoris'haag M, Maguluri GN, Umino Y, Cusato K (2008). Monitoring mouse retinal degeneration with high-resolution spectral-domain optical coherence tomography.. J Vis.

[34] Ruggeri M, Wehbe H, Jiao S, Gregori G, Jockovich ME (2007). In vivo three-dimensional high-resolution imaging of rodent retina with spectral-domain optical coherence tomography.. Invest Ophthalmol Vis Sci.

[35] Srinivasan VJ, Ko TH, Wojtkowski M, Carvalho M, Clermont A (2006). Noninvasive volumetric imaging and morphometry of the rodent retina with high-speed, ultrahigh-resolution optical coherence tomography.. Invest Ophthalmol Vis Sci.

[36] Fischer MD, Huber G, Beck SC, Tanimoto N, Muehlfriedel R (2009). Noninvasive, in vivo assessment of mouse retinal structure using optical coherence tomography.. PLoS ONE.

[37] Nakano N, Ikeda HO, Hangai M, Muraoka Y, Toda Y (2011). Longitudinal and Simultaneous Imaging of Retinal Ganglion Cells and Inner Retinal Layers in a Mouse Model of Glaucoma Induced by N-Methyl-D-Aspartate.. Invest Ophthalmol Vis Sci.

[38] Yamauchi Y, Agawa T, Tsukahara R, Kimura K, Yamakawa N (2011). Correlation between high-resolution optical coherence tomography (OCT) images and histopathology in an iodoacetic acid-induced model of retinal degeneration in rabbits.. Br J Ophthalmol.

[39] Li T, Snyder WK, Olsson JE, Dryja TP (1996). Transgenic mice carrying the dominant rhodopsin mutation P347S: evidence for defective vectorial transport of rhodopsin to the outer segments.. Proc Natl Acad Sci U S A.

[40] Aleman TS, Cideciyan AV, Sumaroka A, Windsor EA, Herrera W (2008). Retinal laminar architecture in human retinitis pigmentosa caused by Rhodopsin gene mutations.. Invest Ophthalmol Vis Sci.

[41] Milam AH, Li ZY, Fariss RN (1998). Histopathology of the human retina in retinitis pigmentosa.. Prog Retin Eye Res.

[42] Humayun MS, Prince M, de Juan E, Barron Y, Moskowitz M (1999). Morphometric analysis of the extramacular retina from postmortem eyes with retinitis pigmentosa.. Invest Ophthalmol Vis Sci.

