# Supplementary figures and images for: Real-Time Imaging of Rabbit Retina with Retinal Degeneration by Using Spectral-Domain Optical Coherence Tomography
Source: PLoS One. 2012 Apr 27;7(4):e36135. doi: 10.1371/journal.pone.0036135 (PMC3338600; doi:10.1371/journal.pone.0036135)

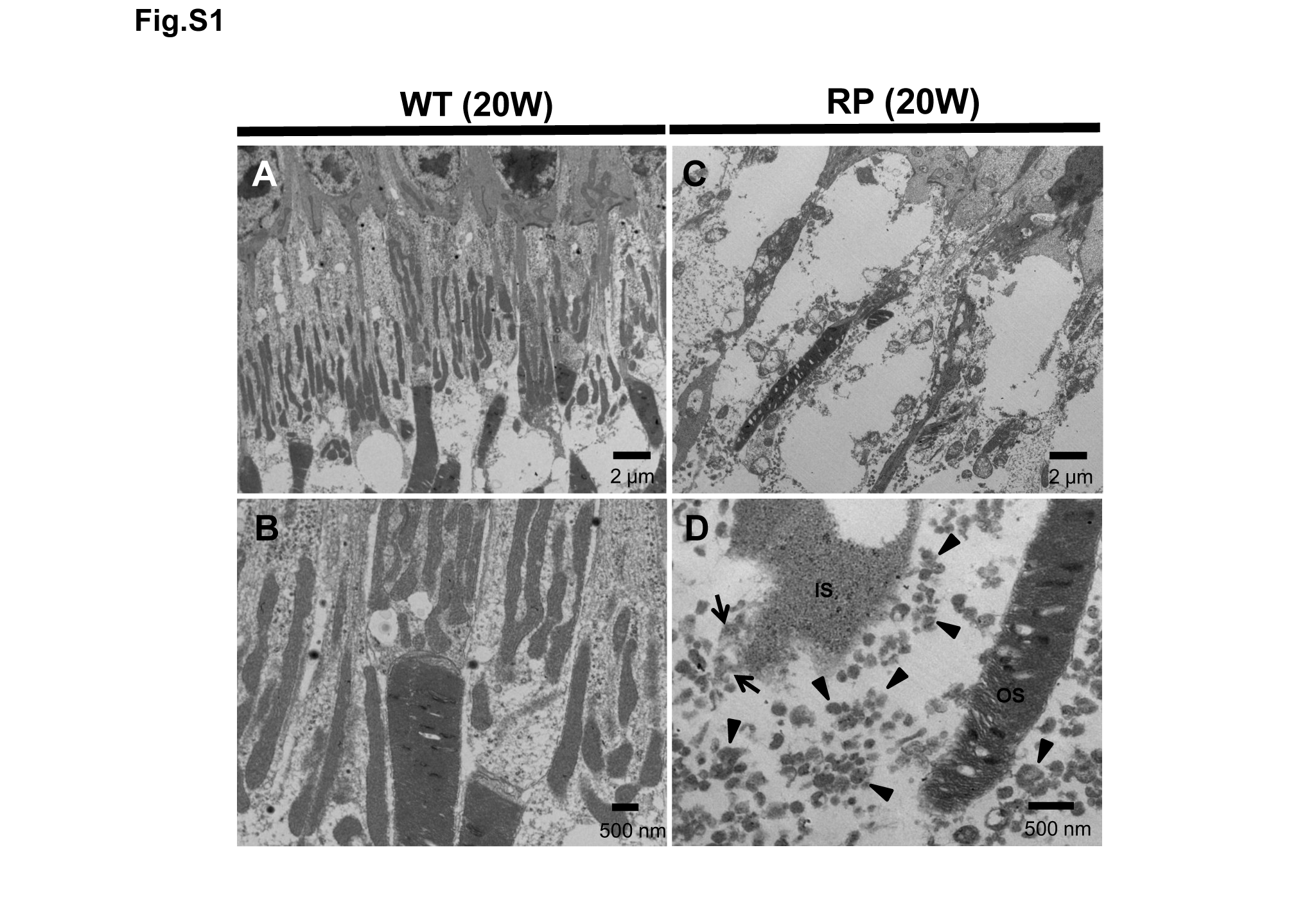

Supplement: Figure S1 — Ultrastructure of photoreceptors in 20-week-old WT and RP rabbits. (A, B) Ultrastructure of photoreceptors in 20-week-old WT rabbits. The inner segments of photoreceptors (IS) and the outer segments of photoreceptors (OS) were regular and dense. (C, D) Ultrastructural changes in 20-week-old RP rabbits. The IS and OS were mostly absent, and the residual IS and OS were less organized than those in WT rabbits. In the magnified image (D), many small vesicles (arrowheads) appeared to be cleaved from the IS into the extracellular space around the photoreceptors (arrows). (TIF) [file pone.0036135.s001.tif]

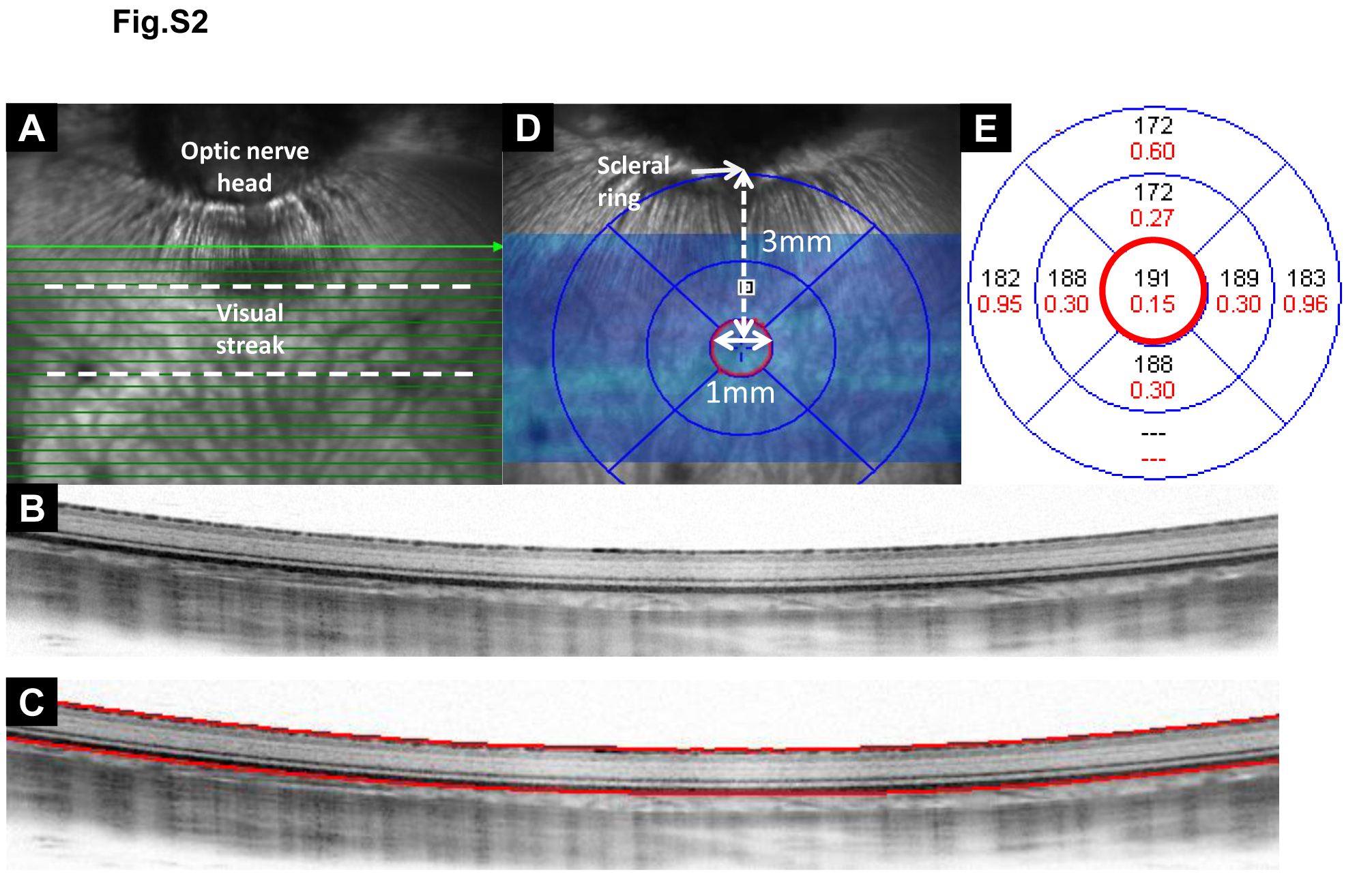

Supplement: Figure S2 — Measurement of mean total retinal thickness. (A) An infrared image on volume scan mode of SD-OCT. In the volume scan mode, the region ventral to the optic disc, including visual streak (19 lines in vertical 15°× horizontal 30°) was imaged. (B) One of the 19 horizontal OCT sections on volume scan mode. The lines of the vitreoretinal interface and the Bruch's membrane are manually delineated at each horizontal section (C). (D) The retinal thickness map constructed from the volume scan OCT images. Total retinal thickness was measured within the red circle shown (E). The diameter of the red circle was 1 mm, and the center was 3 mm ventral to the inferior edge of the ONH (D, E). (TIF) [file pone.0036135.s002.tif]

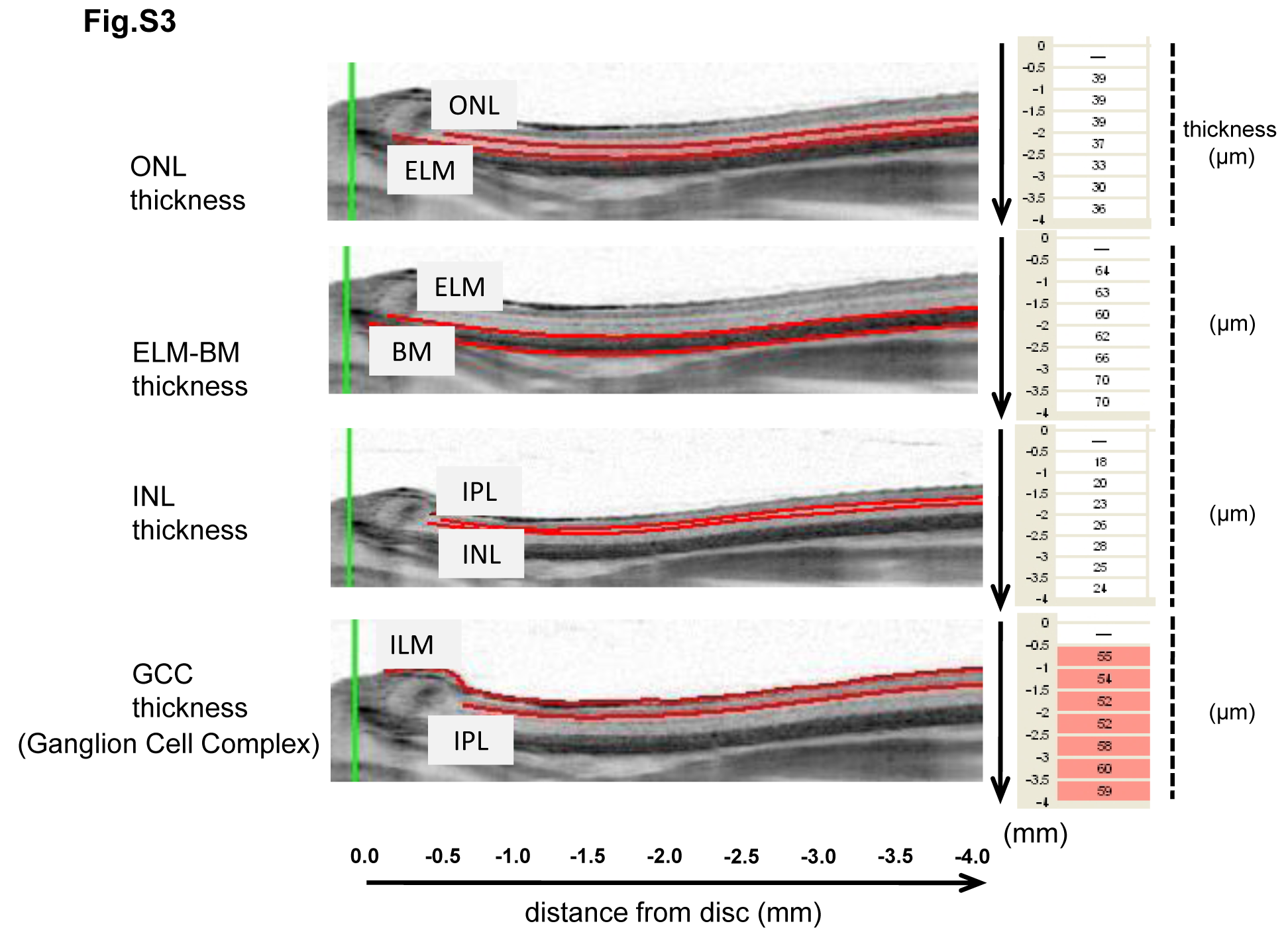

Supplement: Figure S3 — Measurement of the thickness of individual retinal layers. Four vertical OCT sections that pass through the center of the ONH and visual streak are shown. On each section, the boundary lines between each retinal layer were manually delineated. The ONL, ELM–BM, INL, and GCC thicknesses were evaluated in 0.5-mm segments as a function of the distance from the inferior optic disc margin up to 4.0 mm ventral to the inferior edge of the ONH. ONL, outer nuclear layer; ELM, external limiting membrane; BM, Bruch's membrane; INL, inner nuclear layer; and GCC, ganglion cell complex. (TIF) [file pone.0036135.s003.tif]
